# Impact of adjuvant chemotherapy delay on overall survival in early-stage breast cancer: a retrospective analysis

**DOI:** 10.3389/fonc.2025.1666673

**Published:** 2025-09-19

**Authors:** Ahmad Alhalabi, Theresa Abdo, María Herrán, Kaylee Sarna, Rami Tfayli, Zeina Nahleh

**Affiliations:** ^1^ Department of Hematology and Oncology, Maroone Cancer Center, Cleveland Clinic Florida, Weston, FL, United States; ^2^ Center for Clinical Research, Cleveland Clinic Foundation, Cleveland Clinic Florida, Weston, FL, United States

**Keywords:** breast cancer, adjuvant chemotherapy, late start, overall survival, ideal window, real world data

## Abstract

**Background:**

In Early-Stage Breast Cancer (EBC), there is no clear consensus on the ideal window for initiation of adjuvant chemotherapy from the time of definitive surgery. Furthermore, there is a paucity of data on the effectiveness of late chemotherapy. Herein, we aim to assess the effect of delays in adjuvant chemotherapy on overall survival (OS) and to investigate whether there could be a role and remaining effectiveness of late adjuvant chemotherapy compared to no chemotherapy.

**Methods:**

A retrospective cohort study was conducted utilizing data from the National Cancer Database (NCDB), focusing on patients with EBC from 2010 to 2020. Univariate and multivariate Cox regression analyses were employed. Propensity score matching (PSM) (1:1) was performed between the late and no chemotherapy groups to balance baseline characteristics.

**Results:**

N = 326,322 female patients, with a median age of 55 years (Range 47.0-62.0), were identified. Patients were distributed as follows in Group 1 (Adjuvant Chemo < 2 months since surgery N = 266,185, 81.6%), Group 2 (Adjuvant Chemo 2-4 months N = 55,063, 16.9%), Group 3 (Adjuvant Chemo 4-6 months n = 3,749, 1.15%), and Group 4 (Adjuvant Chemo given > 6 months N = 1,325, 0.4%). Multivariate analysis showed that patients in Groups 2,3,4 had worse OS compared to Group 1 (HR 1.28, 95% CI 1.20-1.36, p<0.0001; HR 1.53, 95% CI 1.27-1.84, p<0.0001; and HR 1.46, 95% CI 1.09-1.96, p=0.0104, respectively), indicating that the ideal period to start chemotherapy is within the first 2 months after surgery. When comparing the Late chemotherapy (Group 4) versus a Control Group of patients who declined recommended adjuvant chemotherapy, multivariate analysis indicated that patients in the control group experienced significantly worse OS compared to the late chemotherapy group (HR 1.55, 95% CI 1.13-2.14, p = 0.006).

**Conclusion:**

Systemic adjuvant chemotherapy given within < 2 months period since definitive surgery provides an optimal survival advantage. Notably, patients receiving late adjuvant chemotherapy > 6 months can still derive some benefit compared to those without treatment, making it a viable, though suboptimal, treatment option.

## 1 Introduction

Breast cancer (BC) is the second most common cancer in females after skin cancer, with a lifetime risk of 13.1% (1). In the United States, BC accounts for approximately 300,000 cases each year and is responsible for over 40,000 deaths (2). Management of BC is evolving and requires cooperation among multidisciplinary fields, including surgical intervention, commonly followed by chemotherapy, endocrine therapy, radiation therapy, and/or targeted therapy (3–5). During tumor angiogenesis, the original breast tumor site undergoes vasodilation and an increase in vascular permeability (6). This mechanism is involved in the progression and metastasis of the disease and provides a rationale for subsequent post-operative “adjuvant” systemic therapies (7–10). The benefit of systemic therapy has been well-documented in Early-Stage Breast Cancer (EBC) (11). Meta-analyses by the Early Breast Cancer Trialists’ Collaborative Group (EBCTCG) showed that adjuvant chemotherapy reduces BC mortality and increases survival (12, 13), reinforcing the importance of early intervention and treatment adherence. However, with the growing complexity of health care systems, heightened insurance requirements, and specific patient factors (14), there could be delays in the initiation of systemic treatments (15, 16). Khorana et al. have reported that with each week of delayed adjuvant treatment, there is a 1.2-3.2% increase in mortality (15). Despite numerous studies investigating the optimal timing of adjuvant chemotherapy in EBC, no consensus has been clearly established (12, 17–22). Moreover, large-scale studies evaluating whether delayed initiation of chemotherapy confers survival benefits in this population are lacking.

### 1.1 Objectives

This analysis of the National Cancer Database (NCDB) aims to assess the optimal timing for initiating adjuvant chemotherapy for patients with EBC and investigate whether late chemotherapy (>6months after surgery) can still improve patients’ survival outcomes.

## 2 Methods

This retrospective analysis accessed information from the National Cancer Database (NCDB) between 2010 and 2020 for patients with stages I-III breast cancer, which we will refer to as Early Breast Cancer (EBC) during this analysis. The NCDB is a database that compiles data from over 1,500 Commission on Cancer-accredited facilities across the United States. It contains de-identified, HIPAA-compliant patient-level data and is accessible to investigators from accredited programs. Access to such data is only available via an application process and Participant User Data file (PUF) agreement. The study was conducted after obtaining approval by the Cleveland Clinic’s Institutional Review Board (IRB) as Exempt Human Subject Research (IRB #22-160). The waiver of informed consent to participate and publish the data in an online open-access publication was granted in the application due to minimal risk research involving human subjects. This study was conducted per the regulations of the ethical review committee and the guidelines of the journal.

Records from eligible patients included in this analysis were based on those diagnosed with EBC (Stages I-III) between 2010 and 2020, aged between 18 and 69, who received adjuvant chemotherapy with a known time interval documented from date of definitive surgery. Tumors with all receptor subtypes (Estrogen ER/Progesterone PR/HER2 status and their respective combinations) were included in the analysis. All breast cancer histologies (Invasive ductal, Invasive lobular, and others) were included in the analysis. After applying these inclusion criteria, data from N= 326,322 patients were included in the overall analysis, assessing the timing of chemotherapy, between 0 and more than 6 months after surgery. The following were the exclusion criteria: patients 70 years of age or older and patients younger than 18 years of age. We excluded patients with Metastatic Breast Cancer (Stage IV). We excluded patients with unknown staging, unknown treatment timing, and those who refused chemotherapy.

For the second analysis, which compared late chemotherapy (Patients who received chemotherapy more than 6 months) with controls (Patients who refused chemotherapy), we included data from patients who were offered chemotherapy but declined, forming our control group. N = 48,763 patients were included, and a 1:1 propensity score matching (PSM) was performed, yielding N = 1,466 patients.

The same inclusion-exclusion criteria used for the first analysis were employed; however, we modified them to include our controls.

For both analyses, patients with missing vital status, which indicates whether the patient is alive or deceased, were excluded from the initial data screening. Consequently, approximately 62,300 patients were excluded, resulting in a final sample size of 326,322 patients for the first analysis and 1,466 patients for the second analysis. For other variables with missing data, we conducted our analysis using only the available data; missing values were not used in the analysis.

We excluded a total of 62,300 patients (14.5%) from this study due to missing vital status information, which indicates whether the patient is alive or deceased.

Socio-demographic (age, race, ethnicity, Insurance type, facility type, facility location, urban/rural location, income, and percent of no high school degree) and clinical-pathological characteristics (Days to adjuvant chemotherapy, Charlson-Deyo comorbidity score, histology, stage, grade, estrogen receptor status, progesterone receptor status, HER2 overall summary status, radiotherapy, immunotherapy, regional lymph node status, Oncotype, type of surgery, surgical margins, number of positive nodes, tumor size, and subtype of hormone receptors) were evaluated. For the purpose of this analysis, patients were distributed by time from definitive surgery to adjuvant chemotherapy in groups as follows: Group 1 includes patients initiating chemotherapy within a window of < 2 months. The remaining groups were stratified in an additional 2-month intervals, Group 2 between 2-4 months, Group 3 between 4-6 months, and Group 4 more than 6 months, resulting in four total treatment groups. This classification was based on clinical practice and reports generally supporting the 6-8 weeks window as the ideal timing to initiate adjuvant chemotherapy. Of note, the National Comprehensive Cancer Network does not explicitly recommend a specific timeframe for initiating adjuvant chemotherapy following definitive surgery in EBC. However, guidelines from the European Society of Medical Oncology (ESMO), recommend initiating adjuvant chemotherapy, ideally within 4-6 weeks post-surgery (23). Further literature, such as Gagliato et al. and Kumar et al., supports the 60 days window for ideal survival outcomes (17, 24).

In the second analysis, the late chemotherapy group (Group 4) was compared to a control group of eligible patients who were offered adjuvant chemotherapy but declined it.

Overall survival (OS) was defined as the time in months from the date of diagnosis (time zero) to the date of death or the last known follow-up, as provided by the NCDB’s PUF_Vital_status. Since the exact surgery dates were not known, we calculated the time from surgery to chemotherapy by subtracting “days from diagnosis to surgery” from “days from diagnosis to chemotherapy” and classified them into the four previously mentioned treatment groups.

### 2.1 Statistical analysis

Statistical Analysis System (SAS) version 9.4 and R version 4.2.3 was used. PSM 1:1 was performed for the analysis comparing late chemotherapy with the control group. Chi-square, Fisher’s exact, independent t, and Mann-Whitney U tests were performed to evaluate the association between each categorical characteristic variable. Kaplan-Meier analyses and log-rank tests were performed on the whole dataset and the subgroup analyses. Univariate Cox regressions were performed to determine significance and inclusion into multivariate Cox regression. Cramer’s V was utilized to assess correlation and exclude variables from multivariate Cox regression if a strong association (>0.6) was found. A multivariate Cox regression model was performed using a backward elimination approach with a 0.05 cutoff.

## 3 Results

### 3.1 Sociodemographic and clinical characteristics

We identified N = 326,322 patients who had EBC and were eligible for the first analysis (chemotherapy within 6 + months of surgery). Patients were distributed into Group 1 (n = 266,185, 81.6%), Group 2 (n = 55,063, 16.9%), Group 3 (n = 3,749, 1.15%), and Group 4 (n = 1,325, 0.4%). The cohort consisted of females with a median age of 55 (IQR, 47.0-62.0). Non-Hispanic patients were predominant (88%). Additionally, most of the population had a comorbidity score of zero (84.9%). The stage breakdown was as follows: Stage I (n = 114,511, 44.3%), Stage II (n = 106,963, 41.3%), and Stage III (n = 37,232, 14.4%). These proportions were consistent across all study groups (Groups 1, 2, 3, and 4). There was a predominance of hormone-positive tumors (n = 150,477, 46.2%), followed by Triple-negative tumors (n = 50,407, 15.4%), Triple-positive tumors (n = 37,216, 11.4%) and Hormone receptor-negative, HER2 positive tumors (n = 33,187, 10.2%).

In the second analysis (late chemotherapy), data from N = 42,492 patients who had EBC and received late chemotherapy (Group 4) and Patients who refused chemotherapy (Control Group) were evaluated. After running a 1:1 PSM analysis on age, race, stage, subtypes of hormone receptors, Charlson-Deyo Comorbidity score, and grade, a total of N = 1,466 patients were included in the final analysis, with each group comprising 733 patients. The cohort consisted of females only with a median age of 57 (IQR 48.0-64.0), and a predominance of non-Hispanic patients (82.3%). In addition, most of the population had a comorbidity score of zero (82.1%). The distribution of stages was as follows: Stage 1 (n = 542, 37%), Stage 2 (n = 654, 44.6%), and Stage 3 (n = 270, 18.4%). Furthermore, for the subtypes of hormone receptors, there was a noted predominance of hormone-positive tumors (n = 784, 53.5%), followed by Triple-negative tumors (n = 264, 18%), followed by HER2 positive tumors (n = 229, 15.6%), and lastly Triple-positive tumors (n = 189, 12.9%) (**Tables 1** and **2**).

**Table 1:** Chi-square of baseline sociodemographic and Clinical Characteristics of patients stratified by time to adjuvant chemotherapy, Groups (1–4).

Characteristics, n (%)	Total N = 326,322	Group 1^a^ n=266,185	Group 2^b^ n=55,063	Group 3^c^ n=3,749	Group 4^d^ n=1,325	P -value. Effect size
Age, years, n (%)						<0.001,0.04
≤ 49 ≥ 50	102581 (31.4)	85733 (32.2)	15369 (27.9)	1071 (28.6)	408 (30.8)	
50+ yrs	223741 (68.6)	180452 (67.8)	39694 (72.1)	2678 (71.4)	917 (69.2)	
Charlson Deyo Score, n (%)						<0.001,0.02
0 score	268594 (84.9)	220394 (85.4)	44151 (82.5)	2955 (81.6)	1094 (84.6)	
1 score	38778 (12.3)	30771 (11.9)	7352 (13.7)	491 (13.6)	164 (12.7)	
2 score	6769 (2.1)	5189 (2.0)	1431 (2.7)	127 (3.5)	22 (1.7)	
3+ score	2310 (0.7)	1687 (0.7)	562 (1.1)	48 (1.3)	13 (1.0)	
Ethnicity, n (%)						<0.001,0.03
Hispanic	20622 (6.3)	15540 (5.8)	4546 (8.3)	397 (10.6)	139 (10.5)	
Non-Hispanic	287311 (88.0)	235607 (88.5)	47487 (86.2)	3118 (83.2)	1099 (82.9)	
Unknown	18389 (5.6)	15038 (5.6)	3030 (5.5)	234 (6.2)	87 (6.6)	
Race, n (%)						<0.001,0.02
White	145783 (44.7)	119959 (45.1)	23743 (43.1)	1532 (40.9)	549 (41.4)	
Black	24568 (7.5)	18764 (7.0)	5232 (9.5)	428 (11.4)	144 (10.9)	
Asian	7371 (2.3)	5986 (2.2)	1277 (2.3)	78 (2.1)	30 (2.3)	
Others	148600 (45.5)	121476 (45.6)	24811 (45.1)	1711 (45.6)	602 (45.4)	
Grade, n (%)						<0.001,0.02
Well-differentiated	24892 (8.9)	19787 (8.7)	4658 (9.9)	329 (10.3)	118 (10.4)	
Moderately differentiated	112920 (40.3)	91021 (39.8)	20040 (42.5)	1411 (44.0)	448 (39.5)	
Poorly differentiated/ anaplastic	142234 (50.8)	117759 (51.5)	22439 (47.6)	1469 (45.8)	567 (50.0)	
Histology, n (%)						0.2622,0.004
invasive ductal	265331 (91.1)	216728 (91.1)	44540 (90.9)	2989 (90.4)	1074 (91.0)	
invasive lobular	26007 (8.9)	21127 (8.9)	4458 (9.1)	316 (9.6)	106 (9.0)	
Stage, n (%)						<0.001,0.02
Stage 1	114511 (44.3)	91976 (43.6)	20775 (47.4)	1326 (44.7)	434 (42.3)	
Stage 2	106963 (41.3)	87821 (41.6)	17568 (40.1)	1167 (39.4)	407 (39.7)	
Stage 3	37232 (14.4)	31060 (14.7)	5516 (12.6)	472 (15.9)	184 (18.0)	
Radiotherapy, n (%)						<0.001,0.04
Administered	197825 (60.6)	164545 (61.8)	30551 (55.5)	2015 (53.7)	714 (53.9)	
Not administered	90792 (27.8)	70894 (26.6)	18228 (33.1)	1250 (33.3)	420 (31.7)	
Unknown/others	37705 (11.6)	30746 (11.6)	6284 (11.4)	484 (12.9)	191 (14.4)	
Immunotherapy status, n (%)						<0.001,0.02
Administered	45698 (14.0)	38382 (14.4)	6675 (12.1)	462 (12.3)	179 (13.5)	
Not administered	243463 (74.6)	197504 (74.2)	42200 (76.6)	2811 (75.0)	948 (71.5)	
Unknown	37161 (11.4)	30299 (11.4)	6188 (11.2)	476 (12.7)	198 (14.9)	
Regional lymph nodes status, n (%)						<0.001,0.02
Positive	130308 (39.9)	108082 (40.6)	20285 (36.8)	1421 (37.9)	520 (39.2)	
Negative	155963 (47.8)	125627 (47.2)	27963 (50.8)	1794 (47.9)	579 (43.7)	
Unknown	40051 (12.3)	32476 (12.2)	6815 (12.4)	534 (14.2)	226 (17.1)	
Insurance status, n (%)						<0.001,0.06
Insured	221393 (68.8)	185338 (70.6)	33362 (61.4)	1972 (53.7)	721 (55.6)	
Not insured	7415 (2.3)	5530 (2.1)	1680 (3.1)	156 (4.3)	49 (3.8)	
Governmental	92956 (28.9)	71593 (27.3)	19295 (35.5)	1542 (42.0)	526 (40.6)	
Facility type, n (%)						<0.001,0.02
Community	25289 (9.2)	20457 (9.2)	4357 (9.3)	347 (11.0)	128 (11.7)	
Comprehensive community cancer program	117990 (43.0)	97621 (43.7)	18662 (39.6)	1266 (40.0)	441 (40.5)	
Academic/research program	89350 (32.5)	70708 (31.7)	17136 (36.4)	1102 (34.8)	404 (37.1)	
Integrated Network Cancer Program	42021 (15.3)	34525 (15.5)	6927 (14.7)	452 (14.3)	117 (10.7)	
Urban/rural, n (%)						0.0662,0.01
Rural	3085 (1.6)	2575 (1.6)	463 (1.4)	34 (1.5)	13 (1.6)	
Urban	24326 (12.3)	19995 (12.4)	3958 (12.1)	263 (11.6)	110 (13.6)	
Metro	169728 (86.1)	138676 (86.0)	28400 (86.5)	1965 (86.9)	687 (84.8)	
Oncotype diagnosis recur, n (%)						<0.001,0.06
High risk	31009 (9.5)	22607 (8.5)	7903 (14.4)	402 (10.7)	97 (7.3)	
Intermediate risk	22939 (7.0)	17048 (6.4)	5520 (10.0)	286 (7.6)	85 (6.4)	
Low risk	5465 (1.7)	4194 (1.6)	1133 (2.1)	99 (2.6)	39 (2.9)	
Unknown/others	266909 (81.8)	222336 (83.5)	40507 (73.6)	2962 (79.0)	1104 (83.3)	
Median income quartiles, n (%)						<0.001,0.03
Low income	37771 (16.0)	29428 (15.2)	7521 (19.3)	609 (22.1)	213 (21.0)	
Intermediate low	48852 (20.7)	39731 (20.6)	8264 (21.2)	640 (23.2)	217 (21.4)	
Intermediate high	57971 (24.6)	47838 (24.8)	9269 (23.7)	621 (22.6)	243 (23.9)	
High	91472 (38.7)	76231 (39.5)	14015 (35.9)	883 (32.1)	343 (33.8)	
Type of surgery, n (%)						<0.001,0.02
Lumpectomy	171157 (52.5)	140939 (52.9)	27650 (50.2)	1900 (50.7)	668 (50.4)	
Mastectomy	154966 (47.5)	125092 (47.0)	27379 (49.7)	1840 (49.1)	655 (49.4)	
Surgery not specified/unknown/blanks	199 (0.1)	154 (0.1)	34 (0.1)	9 (0.2)	2 (0.2)	
Surgical margins of primary site, n (%)						<0.001,0.01
No tumor	309653 (94.9)	252807 (95.0)	52153 (94.7)	3466 (92.5)	1227 (92.6)	
Tumor	13779 (4.2)	11069 (4.2)	2415 (4.4)	229 (6.1)	66 (5.0)	
Unknown	2890 (0.9)	2309 (0.9)	495 (0.9)	54 (1.4)	32 (2.4)	
Number of regional nodes positive, n (%)						<0.001,0.03
0 nodes positive	173593 (53.8)	139775 (53.1)	31130 (57.4)	2022 (55.2)	666 (52.1)	
1-3 nodes positive	106195 (32.9)	87797 (33.3)	16901 (31.2)	1096 (29.9)	401 (31.4)	
4 or more positive	42632 (13.2)	35712 (13.6)	6163 (11.4)	545 (14.9)	212 (16.6)	
Tumor size, cm, n (%)						<0.001,0.01
T1	134109 (49.8)	108619 (49.5)	23440 (51.3)	1548 (49.3)	502 (45.2)	
T2	114206 (42.4)	93549 (42.6)	18866 (41.3)	1302 (41.5)	489 (44.0)	
T3	18618 (6.9)	15327 (7.0)	2946 (6.4)	243 (7.7)	102 (9.2)	
T4	2400 (0.9)	1868 (0.9)	466 (1.0)	48 (1.5)	18 (1.6)	
Hormone receptors subtypes n (%)						<0.001,0.03
Triple-negative	50407 (15.4)	42991 (16.2)	6767 (12.3)	481 (12.8)	168 (12.7)	
HER2 Positive	33187 (10)	27688 (10.4)	4959 (9)	374 (10)	166 (12.5)	
Hormone Positive|HER2 Negative	150477 (46)	120685 (45.3)	27383 (50)	1798 (48)	611 (46)	
Triple positive	37216 (11.4)	30883 (11.6)	5757 (10.5)	412 (11.0)	164 (12.4)	
Blanks/unknown	55035 (16.9)	43938 (16.5)	10197 (18.5)	684 (18.2)	216 (16.3)	

_a_: Group 1: Patients who received adjuvant chemotherapy for less than 2 months.

_b_: Group 2: Patients who received adjuvant chemotherapy within 2-4 months.

_c_: Group 3: Patients who received adjuvant chemotherapy within 4-6 months

_d_: Group 4: Patients who received adjuvant chemotherapy more than 6 months

**Table 2:** Chi-square of baseline sociodemographic and Clinical Characteristics of patients receiving late adjuvant chemotherapy (Group 4) vs. patients who refused chemotherapy.

Characteristics, n (%)	Overall (n=1466)	Late Adjuvant Chemotherapy (n=733)	Refused chemotherapy (n=733)	P value
Age, years, n (%)				1.000
≤50	418 (28.5)	209 (28.5)	209 (28.5)	
>50	1048 (71.5)	524 (71.5)	524 (71.5)	
Charlson Deyo Score, n (%)				0.4895
0 score	1204 (82.1)	605 (82.5)	599 (81.7)	
1 score	223 (15.2)	106 (14.5)	117 (16.0)	
2 score	26 (1.8)	13 (1.8)	13 (1.8)	
3+ score	13 (0.9)	9 (1.2)	4 (0.5)	
Ethnicity, n (%)				<0.001
Hispanic	149 (10.2)	70 (9.5)	79 (10.8)	
Not Hispanic	1206 (82.3)	631 (86.1)	575 (78.4)	
Unknown	111 (7.6)	32 (4.4)	79 (10.8)	
Race, n (%)				0.8803
Asian	26 (1.8)	15 (2.0)	11 (1.5)	
Black	171 (11.7)	84 (11.5)	87 (11.9)	
White	628 (42.8)	314 (42.8)	314 (42.8)	
Others	641 (43.7)	320 (43.7)	321 (43.8)	
Grade, n (%)				0.8800
Grade 1	135 (9.2)	70 (9.5)	65 (8.9)	
Grade 2	558 (38.1)	280 (38.2)	278 (37.9)	
Grade 3 and 4	773 (52.7)	383 (52.3)	390 (53.2)	
Histology, n (%)				0.9012
Invasive Ductal	1222 (92.2)	607 (92.2)	615 (92.1)	
Invasive Lobular	104 (7.8)	51 (7.8)	53 (7.9)	
Stage, n (%)				0.6379
Stage 1	542 (37.0)	267 (36.4)	275 (37.5)	
Stage 2	654 (44.6)	324 (44.2)	330 (45.0)	
Stage 3	270 (18.4)	142 (19.4)	128 (17.5)	
Radiotherapy, n (%)				<0.001
Radiotherapy Administered	763 (52.0)	439 (59.9)	324 (44.2)	
Radiotherapy not administered	669 (45.6)	276 (37.7)	393 (53.6)	
Unknown/others	34 (2.3)	18 (2.5)	16 (2.2)	
Immunotherapy status, n (%)				<0.001
Immunotherapy administered	104 (7.1)	86 (11.7)	18 (2.5)	
Immunotherapy not administered	1352 (92.2)	638 (87.0)	714 (97.4)	
Unknown	10 (0.7)	9 (1.2)	1 (0.1)	
Regional lymph nodes status, n (%)				0.0001
Negative	807 (55.0)	370 (50.5)	437 (59.6)	
Positive	616 (42.0)	332 (45.3)	284 (38.7)	
Unknown	43 (2.9)	31 (4.2)	12 (1.6)	
Insurance status, n (%)				0.9950
Governmental	612 (42.5)	304 (42.4)	308 (42.7)	
Insured	767 (53.3)	383 (53.4)	384 (53.2)	
Not insured	60 (4.2)	30 (4.2)	30 (4.2)	
Facility type, n (%)				0.0326
Academic/research	475 (34.6)	256 (37.5)	219 (31.7)	
Community	158 (11.5)	85 (12.5)	73 (10.6)	
Comprehensive Community Cancer Program	573 (41.7)	268 (39.3)	305 (44.1)	
Integrated network cancer program	167 (12.2)	73 (10.7)	94 (13.6)	
Urban/rural, n (%)				0.4515
Metro	1109 (86.0)	493 (86.3)	616 (85.8)	
Rural	25 (1.9)	8 (1.4)	17 (2.4)	
Urban	155 (12.0)	70 (12.3)	85 (11.8)	
Oncotype diagnosis recur, n (%)				0.0065
High risk	98 (6.7)	55 (7.5)	43 (5.9)	
Intermediate risk	149 (10.2)	55 (7.5)	94 (12.8)	
Low risk	37 (2.5)	19 (2.6)	18 (2.5)	
Unknown/others	1182 (80.6)	604 (82.4)	578 (78.9)	
Median income quartiles, n (%)				0.4854
High	453 (36.0)	215 (35.1)	238 (37.0)	
Intermediate high	296 (23.5)	148 (24.1)	148 (23.0)	
Intermediate low	290 (23.1)	135 (22.0)	155 (24.1)	
Low income	218 (17.3)	115 (18.8)	103 (16.0)	
Type of surgery, n (%)				0.0002
Lumpectomy	766 (52.3)	348 (47.5)	418 (57.0)	
Mastectomy	699 (47.7)	385 (52.5)	314 (42.8)	
Surgery not specified/blanks	1 (0.1)	0 (0.0)	1 (0.1)	
Surgical margins of primary site, n (%)				0.1604
No tumor	1359 (92.7)	683 (93.2)	676 (92.2)	
Tumor	85 (5.8)	36 (4.9)	49 (6.7)	
Unknown	22 (1.5)	14 (1.9)	8 (1.1)	
Percent no high school degree, n (%)				0.0331
High school	277 (21.0)	161 (24.3)	116 (17.7)	
Intermediate high school	355 (27.0)	169 (25.5)	186 (28.4)	
Intermediate low school	397 (30.2)	191 (28.9)	206 (31.5)	
Low high school	287 (21.8)	141 (21.3)	146 (22.3)	
Number of regional nodes positive, n (%)				0.0164
0 nodes positive	812 (56.9)	375 (53.1)	437 (60.6)	
1-3 nodes positive	412 (28.9)	223 (31.6)	189 (26.2)	
4 or more positive nodes	203 (14.2)	108 (15.3)	95 (13.2)	
Tumor size, cm, n (%)				0.0960
T1	571 (44.4)	242 (43.8)	329 (44.9)	
T2	563 (43.8)	241 (43.6)	322 (43.9)	
T3	117 (9.1)	60 (10.8)	57 (7.8)	
T4	35 (2.7)	10 (1.8)	25 (3.4)	
Hormone receptors subtypes, n (%)				0.8581
Triple-positive	189 (12.9)	97 (13.2)	92 (12.6)	
Hormone Positive | HER2 Negative	784 (53.5)	396 (54.0)	388 (52.9)	
HER2 Positive	229 (15.6)	114 (15.6)	115 (15.7)	
Triple-negative	264 (18.0)	126 (17.2)	138 (18.8)	

### 3.2 Overall survival analysis

**Figure 1:**
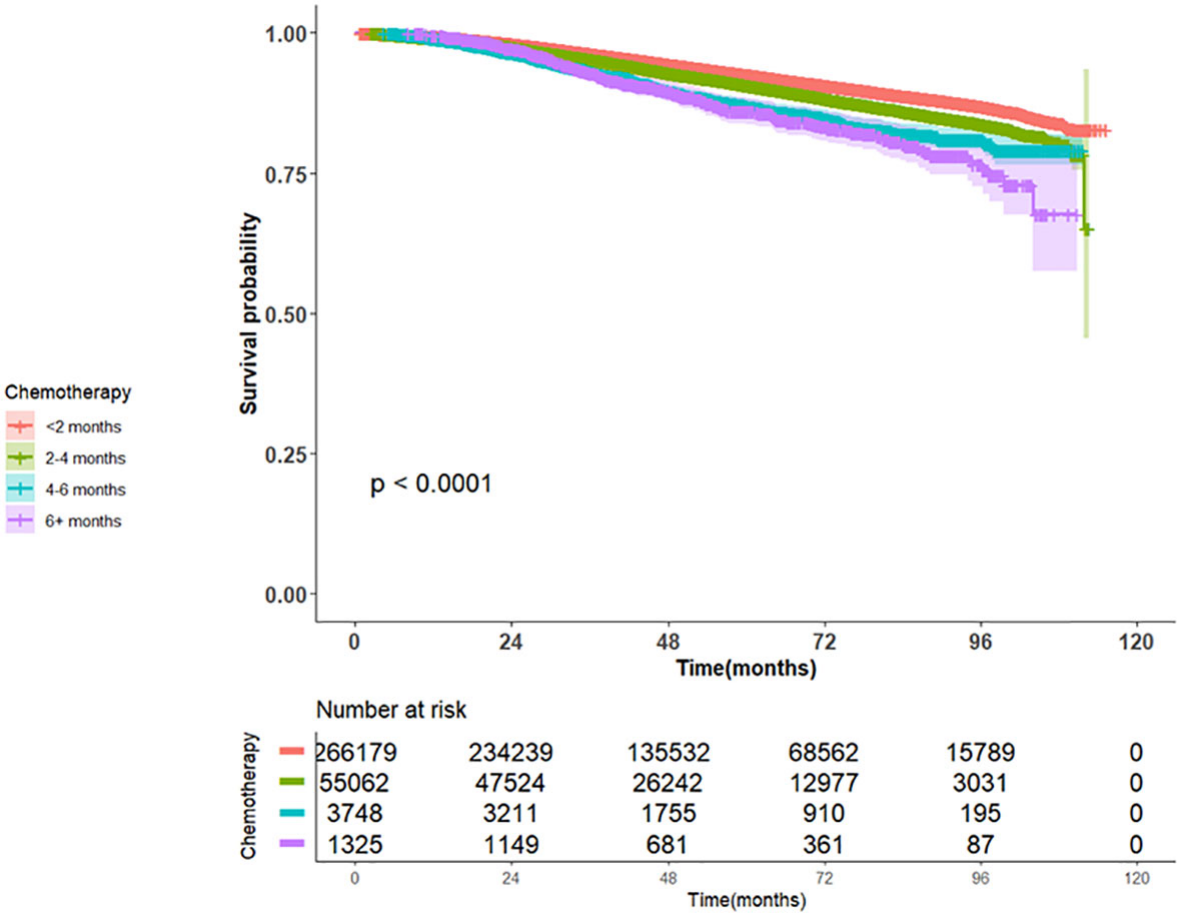
Kaplan Meier Plot of Overall Survival for patients with Early-stage breast cancer stratified by time to adjuvant chemotherapy for Groups 1-4.

Patients in Group 1 (receiving chemotherapy < 2 months since surgery) exhibited the longest OS (45.11 months, 95% CI 44.6-45.7) followed by Group 2 (37.85 months, 95% CI 36.8-38.9), followed by Group 3 (28.94 months, 95% CI 26.9-31.8), and finally Group 4 (30.59 months, 95% CI 27.8-34.3) with a log-rank p-value<0.0001, as reported in **Figure 1** and **Table 3**.

**Table 3:** Overall Survival comparison by time to adjuvant chemotherapy, Groups (1-4).

Days to adjuvant chemotherapy/age	95% OS (months)	95% CI	P-value
< 2 months	45.11	44.6-45.7	
2-4 months	37.85	36.8-38.9	
4-6 months	28.94	26.9-31.8	
6+ months	30.59	27.8-34.3	
Log-rank test			<0.0001

**Table 4** shows the results of multivariable Cox regression, whereby patients in Groups 2,3,4 had an inferior OS compared to Group 1 (HR 1.28, 95% CI 1.20-1.36, p<0.0001, HR 1.53, 95% CI 1.27-1.84, p<0.0001, and HR 1.46, 95% CI 1.09-1.96, p=0.0104, respectively).

**Table 4:** Multivariate Cox Regression Analysis of Clinical and Demographic Predictors of Overall Survival for Groups (1-4):

Variables	Hazard Ratio (95%CI)	p-value
Age: < 50 years(ref.) ≥ 50 years	1.00 1.35(1.27-1.43)	-- <0.0001
Charlson Deyo score 0(ref.) 1 2 3 or more	1.00 1.31(1.23-1.40) 1.81(1.61-2.03) 2.48(2.03-3.02)	-- <0.0001 <0.0001 <0.0001
Ethnicity Hispanic(ref.) Not Hispanic Unknown	1.00 1.61(1.42-1.82) 1.66(1.39-1.98)	-- <0.0001 <0.0001
Race White(ref.) Black Asian Others	1.00 1.03(0.96-1.10) 0.63(0.53-0.76) 0.72(0.65-0.79)	-- 0.4712 <0.0001 <0.0001
Grade 1(ref.) 2 3-4	1.00 1.26(1.12-1.40) 1.81(1.62-2.03)	-- <0.0001 <0.0001
Stage 1(ref.) 2 3	1.00 1.21(1.11-1.30) 2.42(2.22-2.64)	-- <0.0001 <0.0001
Insurance status Insured(ref.) Not insured Governmental	1.00 1.18(1.02-1.37) 1.54(1.46-1.62)	-- 0.0274 <0.0001
Facility type Community Comprehensive community cancer program Academic/research(ref.) Integrated network cancer program	1.19(1.09-1.30) 1.12(1.06-1.19) 1.00 1.11(1.03-1.20)	<0.0001 0.0002 -- 0.0102
Oncotypes Low risk(ref.) Intermediate risk High risk Unknown/others	1.00 0.97(0.73-1.28) 1.43(1.09-1.86) 1.33(1.03-1.72)	-- 0.8215 0.0092 0.0263
Median Income Low Intermediate low Intermediate high High(ref.)	1.17(1.06-1.29) 1.19(1.10-1.29) 1.08(1.00-1.16) 1.00	0.0022 <0.0001 0.0501 --
Type of surgery Lumpectomy(ref.) Mastectomy Surgery not specified/blanks	1.00 1.16(1.10-1.22) 1.12(0.36-3.48)	-- <0.0001 0.8440
Surgical margins No tumor(ref.) Tumor Unknown	1.00 1.41(1.27-1.56) 1.29(0.98-1.71)	-- <0.0001 0.0746
Percent no high school degree Low(ref.) Intermediate low Intermediate high High	1.00 1.03(0.96-1.11) 1.11(1.02-1.22) 1.14(1.03-1.27)	-- 0.4513 0.0154 0.0132
Tumor size T1(ref.) T2 T3 T4	1.00 1.34(1.25-1.43) 1.46(1.32-1.61) 1.81(1.53-2.14)	-- <0.0001 <0.0001 <0.0001
Hormone receptor subtype update Hormone positive(ref.) HER2 Positive Triple-negative Triple positive	1.00 0.86(0.79-0.94) 1.68(1.57-1.78) 0.73(0.67-0.81)	-- 0.0010 <0.0001 <0.0001
Days to adjuvant chemotherapy <2 months(ref.) 2-4 months 4-6 months >6 months	1.00 1.28(1.20-1.36) 1.53(1.27-1.84) 1.46(1.09-1.96)	-- <0.0001 <0.0001 0.0104

### 3.3 Sensitivity analysis comparing overall survival for patients who received adjuvant chemotherapy < 8 weeks (Group 1) vs. ≥ 8 weeks (Group 2)

To better compare our results with existing literature, we performed a sensitivity analysis by lumping the patients’ groups into two groups. Group 1 (Patients receiving adjuvant chemotherapy within less than 2 months (< 8 weeks)) vs. Group 2 (Patients who received adjuvant chemotherapy ≥ 2 months (≥8 weeks)). Patients who received adjuvant chemotherapy ≥ 8 weeks had a statistically significant increased risk of death by 30% compared to group 1. (HR 1.3 95% CI 1.23-1.38 and P value <0.0001).

**Figure 2:**
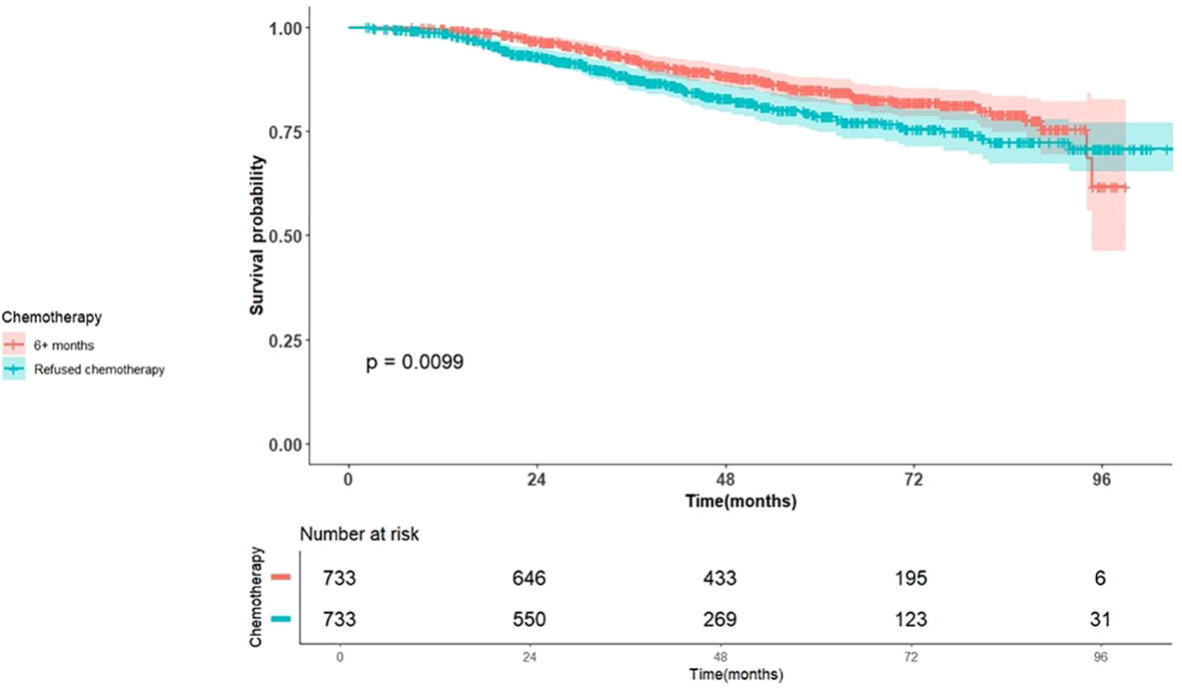
Kaplan Meier Plot of Overall Survival for patients with Early stage breast cancer stratified by chemotherapy, late vs. declined.

The late Chemotherapy group receiving chemotherapy > 6 months (Group 4) exhibited better OS (29.63 months, 95% CI 26.8-34.7) compared to the Control group declining chemotherapy (19.48 months, 95% CI 17.2-24.8, p-value =0.0099), as reported in **Figure 2**.

**Table 5** presents the results of the multivariable Cox regression analysis. Patients in the control group had significantly worse OS compared to the late chemotherapy group (HR 1.55, 95% CI 1.13-2.14, p = 0.006). This survival difference remained consistent across all breast cancer subtypes.

**Table 5:** Multivariate Cox Regression Analysis of Clinical and Demographic Predictors of Overall Survival for Late Chemotherapy vs. Control Group.

Variables	Hazard Ratio (95%CI)	p-value
Charlson Deyo score 0(ref.) 1 2 3 or more	1.00 1.55(1.06-2.25) 2.93(1.36-6.34) 4.40(1.87-10.35)	-- 0.0230 0.0062 0.0007
Grade 1(ref.) 2 3/4	1.00 2.31(0.97-5.48) 3.62(1.55-8.48)	-- 0.0587 0.0030
Stage 1(ref.) 2 3	1.00 1.79(1.21-2.64) 4.21(2.75-6.44)	-- 0.0034 <0.0001
Radiotherapy Radiotherapy(ref.) No radiotherapy Unknown/others	1.00 1.41(1.03-1.93) 2.74(1.24-6.05)	-- 0.0329 0.0128
Insurance status Insured(ref.) Not insured Governmental	1.00 1.47(0.67-3.21) 1.51(1.10-2.07)	-- 0.3349 0.0104
Immunotherapy Immunotherapy(ref.) No immunotherapy Unknown	1.00 1.17(0.55-2.50) 4.14(1.20-14.28)	-- 0.6858 0.0243
Hormone receptor subtype Hormone positive(ref.) HER2 Positive Triple-negative Triple positive	1.00 1.42(0.91-2.19) 1.98(1.34-2.93) 1.36(0.81-2.29)	-- 0.1196 0.0006 0.2445
Chemotherapy group Refused chemotherapy 6+ months(ref.)	1.55(1.13-2.14) 1.00	0.0066 --

Patients in Groups (1–4) who were ≥ 50 years of age exhibited a significantly worse OS compared to patients < 50 years of age (HR 1.35, 95% CI 1.27-1.43, p < 0.0001).

In the analysis of Groups 1-4, as expected, increasing comorbidity scores and higher tumor stages were significantly associated with poorer OS compared to score zero and stage 1. The Hazard Ratio (HR) for comorbidity scores 1,2,3+ compared to score 0 were 1.31 (95% CI 1.23-1.4, p<0.0001), 1.81 (95% CI 1.61-2.03, p<0.0001), and 2.48 (95% CI 2.03-3.02, p<0.0001), respectively. In the late chemotherapy vs. Control groups, increasing comorbidity scores and higher tumor stages were significantly associated with poorer OS compared to score zero and stage 1the HRs for comorbidity scores 1,2,3+ compared to score zero were 1.55 (95% CI 1.06-2.25, p=0.0230), 2.93 (95% CI 1.36-6.34, p=0.0062), and 4.40 (95% CI 1.87-10.35, p=0.0007), respectively. Similarly, the higher tumor stages were associated with worse survival. In Groups 1-4, the HRs for stages 2 and 3 compared to stage 1 were 1.21 (95% CI, 1.11-1.30; p < 0.0001) and 2.42 (95% CI, 2.22-2.64; p < 0.0001), respectively. Furthermore, in the late chemotherapy versus control groups, the HR was 1.79 (95% CI, 1.21-2.64, p = 0.0034) for stage 2 and 4.21 (95% CI, 2.75-6.44, p < 0.0001) for stage 3.

**Figure 3:**
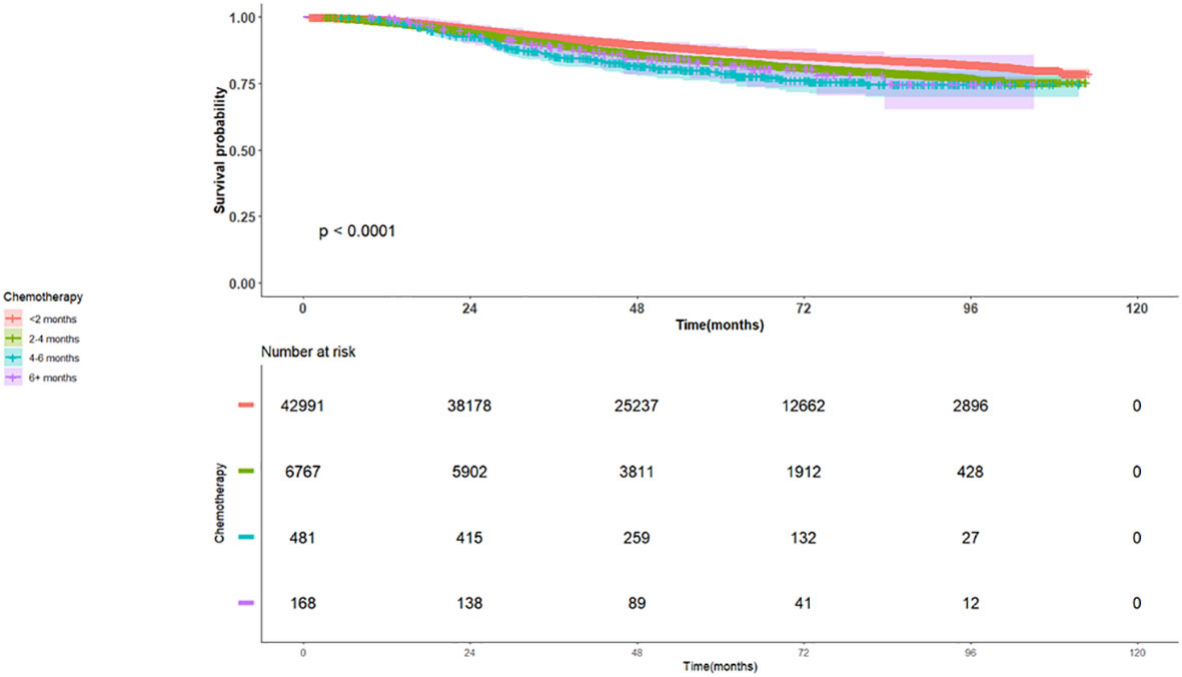
Kaplan Meier Plot of Overall Survival for patients with TNBC early stage breast cancer stratified by time to adjuvant chemotherapy for Groups 1-4.

Regarding breast cancer subtypes, within Groups 1-4, patients with HER2-positive or Triple-Positive tumors demonstrated improved survival compared to those with Hormone- receptor positive tumors (HR 0.86, 95% CI 0.79-0.94, p=0.001; and HR 0.73, 95% CI 0.67-0.81, p<0.0001, respectively). In contrast, patients with triple-negative breast cancer tumors exhibited significantly worse OS (HR 1.68, 95% CI 1.57-1.78, p<0.0001), as illustrated in **Figure 3**.

### 3.4 Subgroup analysis for HER2-positive tumors

We have compared patients with HER2-positive tumors and examined overall survival based on the timing of adjuvant chemotherapy, categorizing them into four well-characterized groups **Table 6**. Patients with HER2-positive breast cancer who received adjuvant chemotherapy within groups 2,3,4 had an inferior OS compared to Group 1 (HR 1.48, 95% CI 1.22-1.79, p<0.0001, HR 1.48, 95% CI 0.76-2.88, p-value = 0.25, and HR 1.05, 95% CI 0.39-2.84, p-value= 0.9162 respectively).

**Table 6:** Multivariate Cox Regression Analysis of HER2 positive and Triple Negative Breast cancer.

Variables	Hazard Ratio (95%CI)	p-value
Days to Adjuvant chemotherapy (HER2 Positive) < 2 months (Ref) (1822/27688) 2-4 months (464/4959) 4-6 months (49/374) >6 months (26/166)	1 1.48 (1.22-1.79) 1.48 (0.76-2.88) 1.05 (0.39-2.84)	-- <0.0001 0.2488 0.9162
Days to Adjuvant Chemotherapy (TNBC) < 2 months (Ref) (5102/42991) 2-4 months (1054/6767) 4-6 months (93/481) >6 months (27/168)	1 1.16 (1.04-1.30) 1.43 (1.01-2.01) 1.63 (0.96-2.77)	-- 0.0110 0.0414 0.0687

### 3.5 Subgroup analysis for triple negative tumors

Patients who received adjuvant chemotherapy within groups 2,3,4 had an inferior OS compared to Group 1 (HR 1.16, 95% CI 1.04-1.3, and a p-value 0.0110, HR 1.43, 95% CI 1.01-2.01, p-value 0.0414, and HR 1.63, 95% CI 0.96-2.77, p-value = 0.0687) **Table 6**.

It is important to note that the number of patients in each group for HER2-positive and triple-negative tumors decreased within each group, and as such, could be a reason for not achieving significant results.In the matched analysis, patients with triple positive tumors and patients with HER-2 positive tumors did not have a significant overall survival difference when compared to patients with Hormone Positive tumors [HR 1.36, 95% CI 0.81-2.29, p=0.2445 and HR 1.42, 95% CI 0.91-2.19, p=0.1196, respectively]. However, patients with triple-negative breast tumors exhibited significantly inferior OS compared to those with Hormone-Positive tumors (HR 1.98, 95% CI 1.34-2.93, p=0.0006).

## 4 Discussion

This analysis reinforces that starting adjuvant chemotherapy within <2 months (< 8 weeks) from definitive surgery provides the optimal survival advantage in EBC. Interestingly, data suggests that patients could still benefit from late chemotherapy compared to no chemotherapy when it is indicated, even if initiation occurs more than 6 months after surgery, for any possible reason. This extensive retrospective analysis exploring OS based on the timing of adjuvant chemotherapy administration as well as the role of late chemotherapy in EBC adds evidence that could contribute to more optimization of clinical practice in EBC, as there has been, thus far, no clear consensus on the optimal time to deliver adjuvant chemotherapy. In addition to that, this analysis provides more granular and in-depth data for the OS based on the time range created for these four groups. In addition, this study provides practical implications and helps answer the clinical question of “to give or not to give” systemic adjuvant chemotherapy for breast cancer patients who present many months beyond their definitive surgery. In clinical practice, around 6 weeks has been considered a generally suitable threshold to provide adjuvant chemotherapy for patients with EBC (17, 18, 23, 25–28). Our study aligns with this timeframe for delivery of adjuvant chemotherapy, as it suggests that an optimal window of <2 months is ideal for the best outcome. Further literature demonstrated that patients with breast-conserving surgery should start adjuvant chemotherapy within 30 days; however, this might not apply to patients with mastectomy (29). On the other hand, some studies recommend more flexible timing thresholds, i.e., initiating adjuvant chemotherapy up to 12 - 16 weeks would be acceptable, beyond which OS would be compromised (12, 19, 20). Also, some studies have not suggested significant survival differences between early and late initiation of adjuvant chemotherapy (30, 31). These differences in outcomes in existing literature may be due to variations in sample size, cohort characteristics, and analytic approaches across the studies.

This analysis is also consistent with other studies reporting on survival outcomes based on breast cancer subtypes. Patients with triple-negative tumors have been reported to exhibit inferior survival outcomes if chemotherapy was initiated beyond a 1-month duration, adding a stricter time frame (32–35). A study by Li S et al. concurs with this finding and adds that this is also true for patients with positive lymph node infiltration (36). Interestingly, while some reports did not clearly demonstrate a significant association with survival in patients receiving chemotherapy for operable TNBC (37), numerous articles have, in contrast, indicated the impact of chemotherapy timing on the OS of TNBC (34, 38). TNBC should be considered an aggressive tumor with an inferior prognosis compared to other subtypes, which necessitates early intervention compared to other subtypes, as it has been associated with a high relapse rate and distal metastasis (38–43). Our analysis consistently established that patients, including those with TNBC, who received adjuvant chemotherapy within less than 2 months demonstrated a clear survival advantage compared to receiving treatment at later time intervals.

Importantly, this study offers a possible option for patients who underwent surgery but, for various reasons, did not receive systemic chemotherapy within 6 months. The data analyzed suggested that adjuvant chemotherapy offers a survival benefit even if the patient receives adjuvant chemotherapy late within their treatment timeline. This is plausible since adjuvant chemotherapy targets microscopic disease systemically, although the earlier chemotherapy is administered, the better the clinical outcomes (44). This is consistent with the treatment of cancer across different solid tumors. For example, patients with pancreatic cancer and locally advanced bladder cancer can still benefit from late systemic adjuvant therapy (45, 46).

To our knowledge, this study is among the first to compare head-to-head late chemotherapy to no chemotherapy in EBC, providing evidence that even late treatment can improve survival outcomes. Furthermore, this study could offer practical implications for patient care, particularly for patients who require additional time to recover post-surgery or following surgical complications. Multiple studies have emphasized the importance of adhering to enhanced recovery after surgery (ERAS) protocol, which leads to better oncologic outcomes (47, 48). Furthermore, healthcare providers might offer comprehensive counseling to address patient hesitancy, facilitating informed decision-making regarding adjuvant chemotherapy; with proper patient education about the disease and its course, there is a significantly improved outcome in terms of quality of life, depression, side effects, and performance status (49, 50).

This study has several strengths. It is based on a large real- world Data from the NCDB, an extensive, nationally representative registry with standardized data collection. The inclusion of over 300,000 patients provides statistical power and enhances confidence in the wide applicability of the results. In the analysis, propensity score matching and multivariable regression were used to adjust for confounding and strengthen internal validity. However, limitations inherent to the retrospective nature must be acknowledged. Selection bias and unmeasured confounders are possible, as the NCDB lacks variables such as performance status, disease progression, recurrence, patient preference, reasons for treatment delay, access to healthcare, variations in treatment protocols (including chemotherapy agents and dosing), and toxicities. Also, survival metrics other than OS such as disease specific survival were not reported.The time to adjuvant chemotherapy was accurately obtained, the OS is measured from diagnosis to last follow-up or death, rather than from surgery date, introducing a slight misalignment between exposure (timing of chemotherapy) and the outcome (OS), since patients might have experienced varying intervals between diagnosis and surgery. Also, in the second analysis of late chemotherapy versus control, NCDB does not track disease progression after diagnosis; therefore, it is possible that some patients in group 4 (Chemotherapy> 6 months) may have experienced progression before undergoing systematic therapy. This could influence survival comparisons with the control group. Finally, it is essential to recognize the differences in healthcare systems and access to care, as these factors have substantial implications for overall survival, i.e., differences exist between the U.S. healthcare system, the European healthcare system, and other global models that govern these systems.

## 5 Conclusion

This large analysis provides real-time data on the benefit of optimal timing of systemic adjuvant chemotherapy and suggests that it is within 2 months from surgery, which appears to confer a survival advantage compared to other delayed time frames. However, patients could continue to benefit from receiving indicated adjuvant systemic therapy at any time interval since surgery, even later than 6 months. Therefore, late adjuvant chemotherapy should be considered a viable treatment option for patients with EBC who had delays for various reasons. Ultimately, the decision to administer adjuvant chemotherapy, including late chemotherapy, should be a shared decision between patients and physicians, and should include a discussion about its toxicity, side effects, and expected benefits, aligning with the patient’s needs and treatment goals.

## Data Availability

The datasets presented in this article are not readily available because the data supporting this study’s findings are available for investigators who are granted access to the National Cancer Database through an online application from the ACS website (https://www.facs.org/quality-programs/cancer-programs/national-cancer-database/puf/). The participant user files (PUF) for the Breast Cancer database were downloaded by the principal investigator associated with a Commission on Cancer (CoC) accredited institution. Requests to access the datasets should be directed to https://www.facs.org/quality-programs/cancer-programs/national-cancer-database/puf/.

## References

[1] Breast cancer facts & Figures: american cancer society; 2024-2025 . Available online at: https://www.cancer.org/content/dam/cancer-org/research/cancer-facts-and-statistics/breast-cancer-facts-and-figures/2024/breast-cancer-facts-and-figures-2024.pdf (Accessed August 26, 2025).

[2] Cancer facts and figures 2025: american cancer society (2025). Available online at: https://www.cancer.org/cancer/types/breast-cancer/about/how-common-is-breast-cancer.html:~:text=About%20310%2C720%20new%20cases%20of%20invasive%20breast%20cancer%20will%20be%20diagnosed%20in%20women (Accessed August 26, 2025).

[3] Chew HK. Adjuvant therapy for breast cancer: who should get what? West J Med. (2001) 174:284–7. doi: 10.1136/ewjm.174.4.284, PMID: 11290691 PMC1071360

[4] Ben-Dror J Shalamov M Sonnenblick A. The history of early breast cancer treatment. Genes (Basel). (2022) 13. doi: 10.3390/genes13060960, PMID: 35741721 PMC9222657

[5] Wang J Wu SG. Breast cancer: an overview of current therapeutic strategies, challenge, and perspectives. Breast Cancer (Dove Med Press). (2023) 15:721–30. doi: 10.2147/BCTT.S432526, PMID: 37881514 PMC10596062

[6] Gunduz N Fisher B Saffer EA. Effect of surgical removal on the growth and kinetics of residual tumor. Cancer Res. (1979) 39:3861–5., PMID: 476622

[7] Madu CO Wang S Madu CO Lu Y. Angiogenesis in breast cancer progression, diagnosis, and treatment. J Cancer. (2020) 11:4474–94. doi: 10.7150/jca.44313, PMID: 32489466 PMC7255381

[8] Zarychta E Ruszkowska-Ciastek B. Cooperation between angiogenesis, vasculogenesis, chemotaxis, and coagulation in breast cancer metastases development: pathophysiological point of view. Biomedicines. (2022) 10. doi: 10.3390/biomedicines10020300, PMID: 35203510 PMC8869468

[9] Ayoub NM Jaradat SK Al-Shami KM Alkhalifa AE. Targeting angiogenesis in breast cancer: current evidence and future perspectives of novel anti-angiogenic approaches. Front Pharmacol. (2022) 13:838133. doi: 10.3389/fphar.2022.838133, PMID: 35281942 PMC8913593

[10] Boudreau N Myers C. Breast cancer-induced angiogenesis: multiple mechanisms and the role of the microenvironment. Breast Cancer Res. (2003) 5:140–6. doi: 10.1186/bcr589, PMID: 12793895 PMC165004

[11] Asselain B Barlow W Bartlett J Bergh J Bergsten-Nordström E Bliss J. Long-term outcomes for neoadjuvant versus adjuvant chemotherapy in early breast cancer: meta-analysis of individual patient data from ten randomised trials. Lancet Oncol. (2018) 19:27–39. doi: 10.1016/S1470-2045(17)30777-5, PMID: 29242041 PMC5757427

[12] Lohrisch C Paltiel C Gelmon K Speers C Taylor S Barnett J. Impact on survival of time from definitive surgery to initiation of adjuvant chemotherapy for early-stage breast cancer. J Clin Oncol. (2006) 24:4888–94. doi: 10.1200/JCO.2005.01.6089, PMID: 17015884

[13] EBCTCG. Effects of chemotherapy and hormonal therapy for early breast cancer on recurrence and 15-year survival: an overview of the randomised trials. Lancet. (2005) 365:1687–717. doi: 10.1016/S0140-6736(05)66544-0, PMID: 15894097

[14] Losk K Vaz-Luis I Camuso K Batista R Lloyd M Tukenmez M. Factors associated with delays in chemotherapy initiation among patients with breast cancer at a comprehensive cancer center. J Natl Compr Canc Netw. (2016) 14:1519–26. doi: 10.6004/jnccn.2016.0163, PMID: 27956536

[15] Khorana AA Tullio K Elson P Pennell NA Grobmyer SR Kalady MF. Time to initial cancer treatment in the United States and association with survival over time: An observational study. PloS One. (2019) 14:e0213209. doi: 10.1371/journal.pone.0213209, PMID: 30822350 PMC6396925

[16] Smith KT Monti D Mir N Peters E Tipirneni R Politi MC. Access is necessary but not sufficient: factors influencing delay and avoidance of health care services. MDM Policy Pract. (2018) 3:2381468318760298. doi: 10.1177/2381468318760298, PMID: 30288438 PMC6125037

[17] Ashok Kumar P Paulraj S Wang D Huang D Sivapiragasam A. Associated factors and outcomes of delaying adjuvant chemotherapy in breast cancer by biologic subtypes: a National Cancer Database study. J Cancer Res Clin Oncol. (2021) 147:2447–58. doi: 10.1007/s00432-021-03525-6, PMID: 33517468 PMC7847714

[18] Yu KD Huang S Zhang JX Liu GY Shao ZM. Association between delayed initiation of adjuvant CMF or anthracycline-based chemotherapy and survival in breast cancer: a systematic review and meta-analysis. BMC Cancer. (2013) 13:240. doi: 10.1186/1471-2407-13-240, PMID: 23679207 PMC3722097

[19] Chavez-MacGregor M Clarke CA Lichtensztajn DY Giordano SH. Delayed initiation of adjuvant chemotherapy among patients with breast cancer. JAMA Oncol. (2016) 2:322–9. doi: 10.1001/jamaoncol.2015.3856, PMID: 26659132 PMC5920529

[20] Chen SY Tang Y Wang SL Song YW Fang H Wang JY. Timing of chemotherapy and radiotherapy following breast-conserving surgery for early-stage breast cancer: A retrospective analysis. Front Oncol. (2020) 10:571390. doi: 10.3389/fonc.2020.571390, PMID: 33072604 PMC7538693

[21] Colleoni M Bonetti M Coates AS Castiglione-Gertsch M Gelber RD Price K. Early start of adjuvant chemotherapy may improve treatment outcome for premenopausal breast cancer patients with tumors not expressing estrogen receptors. Int Breast Cancer Study Group J Clin Oncol. (2000) 18:584–90. doi: 10.1200/JCO.2000.18.3.584, PMID: 10653873

[22] Jara Sánchez C Ruiz A Martín M Antón A Munárriz B Plazaola A. Influence of timing of initiation of adjuvant chemotherapy over survival in breast cancer: a negative outcome study by the Spanish Breast Cancer Research Group (GEICAM). Breast Cancer Res Treat. (2007) 101:215–23. doi: 10.1007/s10549-006-9282-0, PMID: 16823507

[23] Loibl S André F Bachelot T Barrios CH Bergh J Burstein HJ. Early breast cancer: ESMO Clinical Practice Guideline for diagnosis, treatment and follow-up. Ann Oncol. (2024) 35:159–82. doi: 10.1016/j.annonc.2023.11.016, PMID: 38101773

[24] Gagliato Dde M Gonzalez-Angulo AM Lei X Theriault RL Giordano SH Valero V. Clinical impact of delaying initiation of adjuvant chemotherapy in patients with breast cancer. J Clin Oncol. (2014) 32:735–44. doi: 10.1200/JCO.2013.49.7693, PMID: 24470007 PMC3940536

[25] Raphael MJ Biagi JJ Kong W Mates M Booth CM Mackillop WJ. The relationship between time to initiation of adjuvant chemotherapy and survival in breast cancer: a systematic review and meta-analysis. Breast Cancer Res Treat. (2016) 160:17–28. doi: 10.1007/s10549-016-3960-3, PMID: 27632288

[26] Hanna TP King WD Thibodeau S Jalink M Paulin GA Harvey-Jones E. Mortality due to cancer treatment delay: systematic review and meta-analysis. Bmj. (2020) 371:m4087. doi: 10.1136/bmj.m4087, PMID: 33148535 PMC7610021

[27] Abdel-Rahman O. Impact of timeliness of adjuvant chemotherapy and radiotherapy on the outcomes of breast cancer; a pooled analysis of three clinical trials. Breast. (2018) 38:175–80. doi: 10.1016/j.breast.2018.01.010, PMID: 29432980

[28] Farolfi A Scarpi E Rocca A Mangia A Biglia N Gianni L. Time to initiation of adjuvant chemotherapy in patients with rapidly proliferating early breast cancer. Eur J Cancer. (2015) 51:1874–81. doi: 10.1016/j.ejca.2015.07.003, PMID: 26206258

[29] Heeg E Marang-van de Mheen PJ Van Maaren MC Schreuder K Tollenaar R Siesling S. Association between initiation of adjuvant chemotherapy beyond 30 days after surgery and overall survival among patients with triple-negative breast cancer. Int J Cancer. (2020) 147:152–9. doi: 10.1002/ijc.32788, PMID: 31721193 PMC7317578

[30] Shannon C Ashley S Smith IE. Does timing of adjuvant chemotherapy for early breast cancer influence survival? J Clin Oncol. (2003) 21:3792–7. doi: 10.1200/JCO.2003.01.073, PMID: 14551298

[31] Cold S Düring M Ewertz M Knoop A Møller S. Does timing of adjuvant chemotherapy influence the prognosis after early breast cancer? Results of the Danish Breast Cancer Cooperative Group (DBCG). Br J Cancer. (2005) 93:627–32. doi: 10.1038/sj.bjc.6602734, PMID: 16136052 PMC2361615

[32] Okines AFC Kipps E Irfan T Coakley M Angelis V Asare B. Impact of timing of adjuvant chemothapy for early breast cancer: the Royal Marsden Hospital experience. Br J Cancer. (2021) 125:299–304. doi: 10.1038/s41416-021-01428-4, PMID: 34017085 PMC8292350

[33] Bowcock SJ Shee CD Rassam SM Harper PG. Chemotherapy for cancer patients who present late. Bmj. (2004) 328:1430–2. doi: 10.1136/bmj.328.7453.1430, PMID: 15191983 PMC421791

[34] Hatzipanagiotou ME Pigerl M Gerken M Räpple S Zeltner V Hetterich M. Clinical impact of delaying initiation of adjuvant chemotherapy in patients with early triple negative breast cancer. Breast Cancer Res Treat. (2024) 204:607–15. doi: 10.1007/s10549-023-07207-4, PMID: 38238552 PMC10959785

[35] Zhan QH Fu JQ Fu FM Zhang J Wang C. Survival and time to initiation of adjuvant chemotherapy among breast cancer patients: a systematic review and meta-analysis. Oncotarget. (2018) 9:2739–51. doi: 10.18632/oncotarget.23086, PMID: 29416807 PMC5788675

[36] Li S Ma D Shi HH Yu KD Zhang Q. The effect of delayed adjuvant chemotherapy on relapse of triple-negative breast cancer. J Thorac Dis. (2018) 10:2837–41. doi: 10.21037/jtd.2018.04.94, PMID: 29997947 PMC6006075

[37] Pomponio MK Keele LJ Fox KR Clark AS Matro JM Shulman LN. Does time to adjuvant chemotherapy (TTC) affect outcomes in patients with triple-negative breast cancer? Breast Cancer Res Treat. (2019) 177:137–43. doi: 10.1007/s10549-019-05282-0, PMID: 31119565

[38] Li X Yang J Peng L Sahin AA Huo L Ward KC. Triple-negative breast cancer has worse overall survival and cause-specific survival than non-triple-negative breast cancer. Breast Cancer Res Treat. (2017) 161:279–87. doi: 10.1007/s10549-016-4059-6, PMID: 27888421

[39] Waks AG Winer EP. Breast cancer treatment: A review. Jama. (2019) 321:288–300. doi: 10.1001/jama.2018.19323, PMID: 30667505

[40] Obidiro O Battogtokh G Akala EO. Triple negative breast cancer treatment options and limitations: future outlook. Pharmaceutics. (2023) 15. doi: 10.3390/pharmaceutics15071796, PMID: 37513983 PMC10384267

[41] Lee J. Current treatment landscape for early triple-negative breast cancer (TNBC). J Clin Med. (2023) 12. doi: 10.3390/jcm12041524, PMID: 36836059 PMC9962369

[42] Chaudhuri A Kumar DN Dehari D Patil R Singh S Kumar D. Endorsement of TNBC biomarkers in precision therapy by nanotechnology. Cancers (Basel). (2023) 15. doi: 10.3390/cancers15092661, PMID: 37174125 PMC10177107

[43] Dass SA Tan KL Selva Rajan R Mokhtar NF Mohd Adzmi ER Wan Abdul Rahman WF. Triple negative breast cancer: A review of present and future diagnostic modalities. Medicina (Kaunas). (2021) 57. doi: 10.3390/medicina57010062, PMID: 33445543 PMC7826673

[44] Shien T Iwata H. Adjuvant and neoadjuvant therapy for breast cancer. Jpn J Clin Oncol. (2020) 50:225–9. doi: 10.1093/jjco/hyz213, PMID: 32147701

[45] Mirkin KA Greenleaf EK Hollenbeak CS Wong J. Time to the initiation of adjuvant chemotherapy does not impact survival in patients with resected pancreatic cancer. Cancer. (2016) 122:2979–87. doi: 10.1002/cncr.30163, PMID: 27328270

[46] Corbett CJ Xia L Mamtani R Malkowicz SB Guzzo TJ. Survival benefit persists with delayed initiation of adjuvant chemotherapy following radical cystectomy for locally advanced bladder cancer. Urology. (2019) 132:143–9. doi: 10.1016/j.urology.2019.05.038, PMID: 31199968

[47] Pang Q Duan L Jiang Y Liu H. Oncologic and long-term outcomes of enhanced recovery after surgery in cancer surgeries - a systematic review. World J Surg Oncol. (2021) 19:191. doi: 10.1186/s12957-021-02306-2, PMID: 34187485 PMC8243430

[48] Patient-Reported Outcomes-. and return to intended oncologic therapy after. Ann Surg Open. (2023) 4:0000000000000267. doi: 10.1097/AS9.0000000000000267

[49] Tian J Jia LN Cheng ZC. Relationships between patient knowledge and the severity of side effects, daily nutrient intake, psychological status, and performance status in lung cancer patients. Curr Oncol. (2015) 22:e254–8. doi: 10.3747/co.22.2366, PMID: 26300675 PMC4530822

[50] Periasamy U Mohd-Sidik S Akhtari-Zavare M Rampal L Ismail SIF Mahmud R. Effects of counselling on quality of life among cancer patients in Malaysia: A randomized controlled trial. Iran J Public Health. (2020) 49:1902–11. doi: 10.18502/ijph.v49i10.4693, PMID: 33346212 PMC7719651

